# Identification of proteins regulating phenotype-associated genes of M2 macrophages: a bioinformatic analysis

**DOI:** 10.18699/vjgb-25-104

**Published:** 2025-12

**Authors:** E.A. Antropova, I.V. Yatsyk, P.S. Demenkov, T.V. Ivanisenko, V.A. Ivanisenko

**Affiliations:** Institute of Cytology and Genetics, Siberian Branch of the Russian Academy of Sciences, Novosibirsk, Russia; Institute of Cytology and Genetics, Siberian Branch of the Russian Academy of Sciences, Novosibirsk, Russia; Institute of Cytology and Genetics, Siberian Branch of the Russian Academy of Sciences, Novosibirsk, Russia; Institute of Cytology and Genetics, Siberian Branch of the Russian Academy of Sciences, Novosibirsk, Russia; Institute of Cytology and Genetics, Siberian Branch of the Russian Academy of Sciences, Novosibirsk, Russia

**Keywords:** macrophage phenotypes, expression regulation, proteomes, ANDSystem, automated text analysis, фенотипы макрофагов, регуляция экспрессии, протеомы, система ANDSystem, автоматический анализ текстов

## Abstract

Macrophages are immune system cells that perform various, often opposing, functions in the organism depending on the incoming microenvironment signals. This is possible due to the plasticity of macrophages, which allows them to radically alter their phenotypic characteristics and gene expression profiles, as well as return to their original, non-activated state. Depending on the inductors acting on the cell, macrophages are activated into various functional states. There are five main phenotypes of activated macrophages: M1, M2a, M2b, M2c, and M2d. Although the amount of genome-wide transcriptomic and proteomic data showing differences between major macrophage phenotypes and non-activated macrophages (M0) is rapidly growing, questions regarding the mechanisms regulating gene and protein expression profiles in macrophages of different phenotypes still remain. We compiled lists of proteins associated with the macrophage phenotypes M1, M2a, M2b, M2c, and M2d (phenotype-associated proteins) and analyzed the data on potential mediators of macrophage polarization. Furthermore, using the computational system ANDSystem, we conducted a search and analysis of the relationships between potential regulatory proteins and the genes encoding the proteins associated with the M2 group phenotypes, obtaining estimates of the statistical significance of these relationships. The results indicate that the differences in the M2a, M2b, M2c, and M2d macrophage phenotypes may be attributed to the regulatory effects of the proteins JUN, IL8, NFAC2, CCND1, and YAP1. The expression levels of these proteins vary among the M2 group phenotypes, which in turn leads to different levels of gene expression associated with specific phenotypes.

## Abstract (ru)

Макрофаги – клетки иммунной системы, выполняющие в организме различные, часто противоположные функции в зависимости от поступающих сигналов микроокружения. Это возможно благодаря пластичности макрофагов, позволяющей кардинально менять фенотипические признаки и профили экспрессии генов, а также возвращаться в исходное, неактивированное состояние. В зависимости от действующих на клетку индукторов макрофаги поляризуются в различные функциональные состояния. Принято выделять пять основных фенотипов активированных макрофагов: М1, M2a, M2b, M2c и M2d. Хотя количество полногеномных транскриптомных и протеомных данных, показывающих различия между основными фенотипами макрофагов и неактивированными макрофагами (M0), растет стремительно, все еще остаются вопросы, касающиеся механизмов регуляции профилей экспрессии генов и белков у макрофагов разных фенотипов. Нами были составлены списки белков, ассоциированных с фенотипами макрофагов M1, M2a, M2b, M2c, M2d (фенотип-ассоциированные белки), проанализированы данные о возможных посредниках поляризации макрофагов. Далее с использованием компьютерной системы ANDSystem проведен поиск и анализ связей между потенциальными регуляторными белками и генами, кодирующими белки, ассоциированные с фенотипами группы M2, получены оценки статистической значимости этих связей. Результаты указывают на то, что различия в фенотипах макрофагов М2a, M2b, M2c, M2d могут быть обусловлены регуляторными действиями белков JUN, IL8, NFAC2, CCND1 и YAP1. Уровень их экспрессии варьируется в зависимости от фенотипов группы M2, что в свою очередь приводит к различным уровням экспрессии генов, связанных с конкретными фенотипами

## Introduction

Macrophages are immune system cells that play a key role in processes such as: maintaining body homeostasis (Mosser et al., 2021), defense against infections (Zhang M., Wang, 2014), proinflammatory and anti-inflammatory responses (Xu et al., 2013), tissue regeneration with concomitant stimulation of proliferation (Wynn, Vannella, 2016), and many others. The ability of macrophages to exhibit different functions through polarization (changing their functional state depending on signals from the microenvironment) is associated with their unique plasticity (Mills, 2012; Gurvich et al., 2020). Polarization leads to macrophages acquiring various phenotypes – functional states characterized by unique morphological, molecular and functional features, depending on the polarization inducers: proteins, peptides, polysaccharides, etc

Each macrophage phenotype is characterized by a group of proteins (Martinez et al., 2008). These groups overlap, but different macrophage phenotypes can have radically different functions. For example, the M1 phenotype corresponds to proinflammatory macrophages, essential for the body’s response to infections. M2a macrophages promote wound healing and clear the body of apoptotic cells (Murray et al., 2014). M2b macrophages are called regulatory for their ability to regulate T-helper cells, which leads to a switch in the immune response from proinflammatory to anti-inflammatory. M2c macrophages are necessary for tissue remodeling and the phagocytosis of apoptotic cells. M2d macrophages are called tumor-associated macrophages because they accompany tumor tissues (Zhang Q., Sioud, 2023).

In several studies, a link has been demonstrated between specific macrophage phenotypes and certain pathologies, as well as an association of disease outcomes with particular macrophage phenotypes. For example, patients with ovarian cancer exhibited a pronounced predominance of M1 phenotype macrophages over M2, which was associated with improved survival (Zhang M. et al., 2014). Additionally, the shift of macrophages from the M2 phenotype to M1 suppressed tumor metastasis (Yuan et al., 2017). Research on juvenile idiopathic arthritis in remission showed that the M2 macrophage group predominantly consisted of M2b and M2c, while the number of M2a macrophages was significantly reduced (Feng et al., 2021). In contrast, children with active juvenile idiopathic arthritis had a predominance of M2a and M2b macrophages, while the presence of M2c was decreased. The study of differences between macrophage phenotypes holds significant fundamental importance and also represents substantial practical interest for early disease diagnosis, prognosis, and management of disease progression (Zhang M. et al., 2014; Lampiasi, 2023).

It should be noted that there is conflicting information in the literature regarding the proteins and genes characterizing different macrophage phenotypes. For example, the fractalkine receptor (CX3CR1) is designated as a marker of the M2a phenotype in one publication (Joerink et al., 2011), while in another publication (Chhor et al., 2013), this protein is identified as a marker of the M1 phenotype. Metalloproteinase MMP12 is highlighted as a marker of the M1 phenotype (Hirani et al. 2021), but the article (Lee et al. 2014) shows that this protein is characteristic of the proteomes of the M2 phenotype and dendritic cells. The chemokine CXCL13 is described as an M1 marker in the study (Martinez et al. 2006), while in the work (van der Lans et al. 2015) it is noted as a marker of M2.

How do proteomes intersect in macrophages of different phenotypes to achieve significant functional differences? What molecular and genetic regulatory mechanisms underlie macrophage polarization? Despite the rapid accumulation of genome-wide transcriptomic and proteomic data characterizing the differences between the major macrophage phenotypes and their differences from non-activated macrophages (M0) (Gurvich et al., 2020; Oates et al., 2023), questions about how gene and protein expression profiles are regulated in macrophages of different phenotypes remain open.

The aim of this study was to identify mediator proteins that control the activity of phenotype-associated genes in different phenotypes of M2 macrophages. For this purpose, we used the ANDSystem information system, which is based on machine learning and artificial intelligence methods, including graph neural networks (Ivanisenko V.A. et al., 2015; Ivanisenko T.V. et al., 2024). ANDSystem provides automated analysis of scientific publication texts and factographic databases in the medical and biological domains. Currently, the ANDSystem knowledge base contains knowledge and facts extracted from more than 40 million scientific publications and patents, as well as factual databases, including information on molecular and genetic objects and processes that are important for the functioning of gene networks and their basic components: metabolic networks, signal transduction pathways, DNA-protein and protein-protein interaction networks. The effectiveness of ANDSystem has been demonstrated in a wide range of studies: reconstruction of molecular genetic mechanisms of asthma and hypertension comorbidity (Zolotareva et al., 2019), analysis of the plasma metabolome of patients with postoperative delirium (Ivanisenko V.A. et al., 2023), reconstruction of the hypermethylation regulatory network affecting the development of hepatocellular carcinoma in hepatitis C virus disease (Antropova et al., 2023).

In this work, the following tasks were addressed: 1) formation of phenotype-associated protein lists in macrophages of the main phenotypes (M1, М2a, M2b, M2c, M2d); 2) analysis of differential protein expression data in the M2 phenotype group as potential mediators of macrophage polarization; 3) analysis of regulatory relationships from mediator proteins to genes encoding phenotype-associated proteins using ANDSystem

## Materials and methods

**Proteomic data on macrophages of different phenotypes.** Two types of information about proteins in different macrophage phenotypes were used in the work

1) Our curated database MACRO_GENES, containing lists of genes and proteins associated with macrophage phenotypes (Table S1)1. It was formed through manual analysis of scientific publications describing characteristic proteins that allow differentiation of macrophage phenotypes M1, M2a, M2b, M2c, M2d. Only those proteins, the presence of which in macrophages of certain phenotypes was confirmed by experimental data, were included in the MACRO_GENES database.


Supplementary Materials are available in the online version of the paper: https://vavilov.elpub.ru/jour/manager/files/Suppl_Antropova_Engl_29_7.pdf


2) Proteomic data on differentially expressed proteins in M2a, M2b, M2c, and M2d macrophage phenotypes were obtained from the work by P. Li and colleagues (2022): approximately 200 proteins for each phenotype under consideration. Hereafter, such proteins will be referred to as regulatory proteins or differentially expressed proteins

**Search for potential regulators influencing the activity of phenotype-associated genes. **The search for potential regulatory proteins influencing the activity of phenotype-associated genes was carried out using the knowledge base of the ANDSystem software and the ANDVisio software module included in this system (Demenkov et al., 2012; Ivanisenko V.A. et al., 2015; Ivanisenko T.V. et al., 2024). The ANDSystem knowledge base includes information on interactions between molecular biological objects (genes, proteins, metabolites, biological processes, etc.), obtained through automated analysis of over 40 million scientific publications and patents, as well as a large number of biomedical factual databases. The current version of this knowledge base contains information on over 36 million proteins from various organisms and approximately the same number of genes, 76 thousand metabolites, 100 million interactions, 21 thousand diseases, and more.

**Fig. 1.**
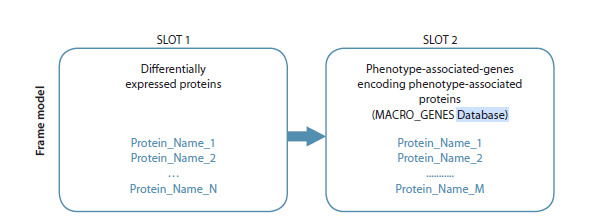
Schematic diagram of a frame model for searching for regulatory links between differentially expressed proteins and phenotype-associated genes.

To search for connections between regulatory proteins and phenotype-associated genes, the frame model software of the ANDSystem was used (Fig. 1). Step 1: The first slot of the frame was filled based on proteomic analysis data (Li et al., 2022) with a list of differentially expressed proteins for each phenotype (M2a, M2b, M2c, and M2d). Step 2: The second slot of the frame was filled with a list of phenotype-associated genes for the same phenotype from our curated MACRO_GENES database (Table S1). Step 3: Using the ANDVisio software module with the filled frame, regulatory connections described in the ANDSystem knowledge base were searched for the studied macrophage phenotype.

The analysis resulted in graphs of regulatory processes, in which nodes corresponded to differentially expressed proteins from the paper (Li et al., 2022) and phenotype-associated macrophage genes from the MACRO_GENES database. Edges connecting graph nodes corresponded to regulatory relationships between them

**Search for functionally significant regulatory proteins of phenotype-associated macrophage genes in regulatory process graphs.** A key step in analyzing regulatory processes, associated with macrophage phenotype-associated genes and identified using frame models, is the search for functionally significant regulatory proteins (also called central nodes). Central nodes play a key role in signaling and coordinating regulatory processes. A wide range of methods have been developed to assess centrality (Ghasemi et al., 2014; Jalili et al., 2016; Ivanisenko V.A. et al., 2019). In our study, node centrality was assessed based on the number of interactions of the protein in question with phenotype-associated genes of the corresponding phenotype.

A high degree of centrality can be observed as a result of functional innovations between genes and proteins, as well as due to random factors. To distinguish between these situations, the statistical significance of the observed degree of centrality was assessed using the hypergeometric test. In this context, the hypergeometric test is used to measure the number of connections between a given protein and randomly determined phenotype-associated genes.

Here: M is the total number of genes represented in the ANDSystem knowledge base; N is the total number of genes with which a specific protein interacts in the ANDSystem knowledge base; n is the number of phenotype-associated genes for a specific phenotype in the MACRO_GENES database; x is the observed number of interactions of the protein in question with phenotype-associated genes for a specific phenotype. Then, under the null hypothesis of a random distribution of interactions, the value of X obeys the hypergeometric distribution law

X ~ Hypergeom(M, N, n),

and the p-value for the right-tailed (enrichment) test was calculated using the formula

**Formula. 1.**
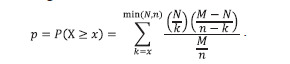
Formula 1

p-values were calculated using the SciPy Python library (scipy.stats.hypergeom). A Bonferroni correction was used to correct for multiple testing. At p < 0.05, the observed degree of centrality was considered statistically significant, and the corresponding protein was considered as a functionally significant regulatory protein controlling the expression of phenotype-associated genes

## Results and discussion

Our work aimed to identify regulatory proteins that influence genes, the expression of which differs between the M2a, M2b, M2c, and M2d macrophage phenotypes. Understanding the regulatory mechanisms that determine differences between macrophage phenotypes is not only of fundamental importance but also holds promise for applications in medicine and pharmacology, as the prevalence of a certain macrophage phenotype has been shown to be associated with the development and outcome of a number of pathologies (Zhang M. et al., 2014; Yuan et al., 2017; Feng et al., 2021).

**General characteristics of phenotype-associated genes and proteins of macrophages M1, M2a, M2b, M2c, M2d**

Table 1 presents a summary of our curated database, MACRO_ GENES, of phenotype-associated genes encoding phenotypeassociated proteins, i. e., proteins specific to macrophages of each of the phenotypes under consideration: M1, M2a, M2b, M2c, and M2d. The presence of proteins in specific phenotypes was confirmed by experimental data presented in the relevant publications. A detailed description of the gene information in MACRO_GENES is given in Table S1.

**Table 1.**
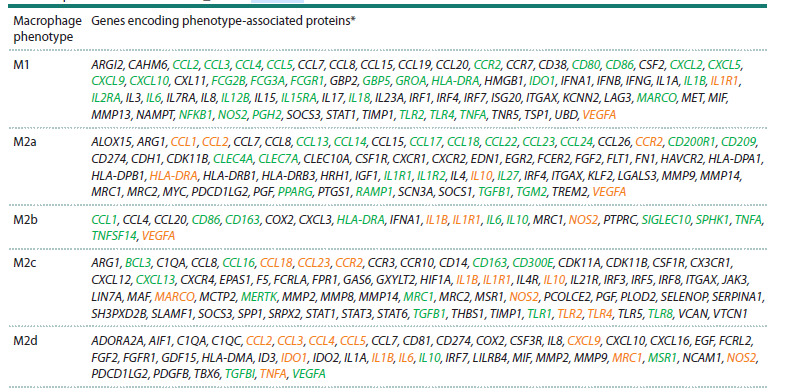
Lists of genes encoding phenotype-associated proteins of macrophages M1, M2a, M2b, M2c and M2d presented in the MACRO_GENES database * Genes encoding proteins that are markers of various macrophages phenotypes are highlighted in green. Genes that are expressed in macrophages of a particular phenotype, according to some sources, but are markers of macrophages of a different phenotype, according to other sources, are highlighted in orange.

Some phenotype-associated proteins are used in experimental studies as specific markers for distinguishing macrophage phenotypes. In Table 1, the genes encoding such proteins are highlighted in green. If a protein is characteristic of a specific phenotype but is also considered a specific marker for another phenotype, the gene encoding it is highlighted in orange (Table 1). For example, the CCL2 protein is considered a marker for the M1 phenotype, but some publications indicate that it is also characteristic of the M2a and M2d phenotypes. Table 1 illustrates the complex pattern of marker intersections between different macrophage phenotypes

**Fig. 2.**
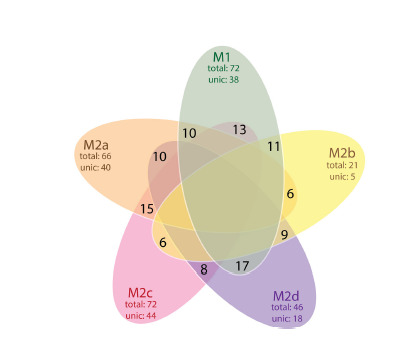
Venn diagram for comparison of macrophage phenotypes M1, M2a, M2b, M2c, M2d according to the gene lists presented in the MACRO_GENES database.

Figure 2 shows a Venn diagram demonstrating the distribution of genes encoding phenotype-associated proteins across five macrophage types (M1, M2a, M2b, M2c, M2d). The diagram is constructed based on the information provided in Table 1. Note that the M1 and M2d phenotypes have the most matching proteins (17). The M2a/M2c and M1/M2c phenotype pairs have 15 and 13 common proteins, respectively. The M2b and M1 phenotypes have 11 matching proteins. A relatively small number of matching proteins (8) can be noted for the M2c and M2d phenotype pair. M2b has the fewest overlaps (6 proteins) with M2a.

**General characteristics of differentially expressed proteins of the M2 macrophage group**

To search for mediator proteins that transmit signals from macrophage polarization inducers to phenotype-associated genes, we used lists of differentially expressed proteins (compared to non-activated macrophages) from P. Li et al. (2022). The published data, summarized in Tables S2 and S3, indicate that the distribution of differentially expressed proteins across the four macrophage phenotypes (M2a, M2b, M2c, M2d) is characterized by significant overlap, i. e., there is no one-to-one correspondence between differentially expressed proteins and macrophage phenotypes. Therefore, to identify regulatory pathways that mediate macrophage polarization into different phenotypes, we required bioinformatic analysis of large volumes of molecular genetic data, conducted using the ANDSystem computer system

**Search for regulatory links from differentially expressed proteins to phenotype-associated genes of macrophages based on frame models**

To analyze large volumes of published data on various macrophage phenotypes, we used the methods and information resources of computer-aided knowledge engineering implemented in the ANDSystem. Using the framework-based approach realized in this system, we searched for regulatory links between differentially expressed proteins and phenotypeassociated genes in macrophages

**Fig. 3.**
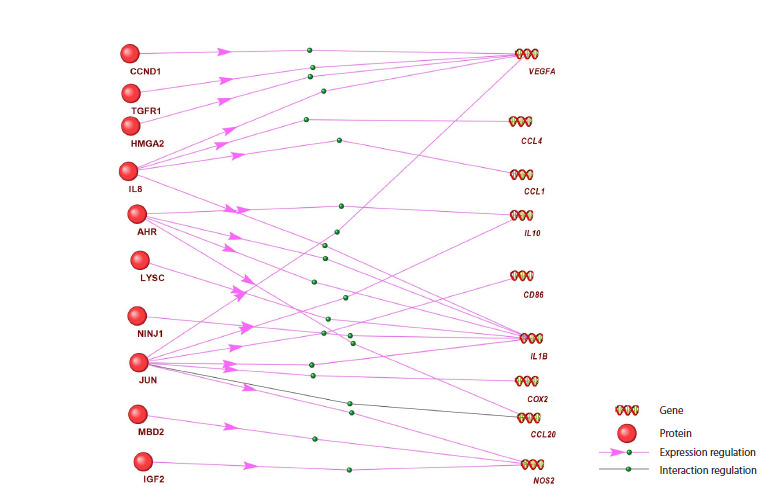
A graph of potential regulatory links between differentially expressed proteins (left) and phenotype-associated genes (right) in M2b macrophages, presented in the ANDSystem interface. Green balls on the arrows in the interactive ANDSystem interface allow users to obtain additional information about specific regulatory links.

Regulatory process graphs were reconstructed, with nodes corresponding to differentially expressed proteins from the paper (Li et al., 2022) and phenotype-associated macrophage genes from the MACRO_GENES database. Edges connecting graph nodes corresponded to regulatory relationships between them. Figure 3 shows an example of a graph of potential regulatory relationships between differentially expressed proteins and phenotype-associated macrophage genes in the M2b phenotype

Figure 3 shows that most phenotype-associated genes are regulated by more than one protein. Furthermore, most of the proteins shown in the figure are involved in the regulation of multiple genes. Similar regulatory relationship diagrams for M2a, M2c, and M2d macrophages are presented in the Supplementary Materials (Fig. S1–S3).

**Identification of statistically significant regulators of phenotype-associated genes**

Quantitative characteristics of regulatory links between differentially expressed proteins and phenotype-associated genes identified using frame models are shown in Section A of Table 2.

**Table 2.**
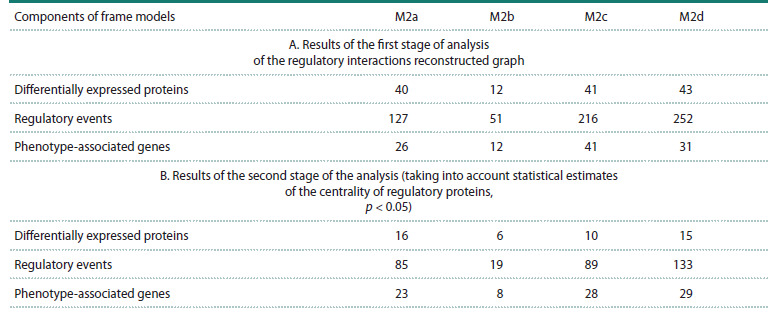
Quantitative characteristics of potential regulatory links identified based on frame models

In the second stage of the analysis, centrality metrics characterizing the functional significance of differentially expressed proteins for the regulation of phenotype-associated genes were assessed. Centrality assessments allowed us to identify proteins regulating phenotype-associated genes with a Bonferroni-corrected statistical significance threshold of p < 0.05 (Table 2B). Accounting for the statistical significance of differentially expressed proteins based on centrality metrics led to a significant reduction in the number of nodes corresponding to phenotype-associated genes and the number of edges corresponding to regulatory events. For example, for the M2a phenotype, of the 40 differentially expressed proteins associated with phenotype-associated genes, 16 were statistically significantly associated with these genes (Table 2B). For the M2b phenotype, it was 6 out of 12 proteins. Accordingly, the number of regulatory events and phenotype-associated genes in the reconstructed graphs decreased.

**Fig. 4.**
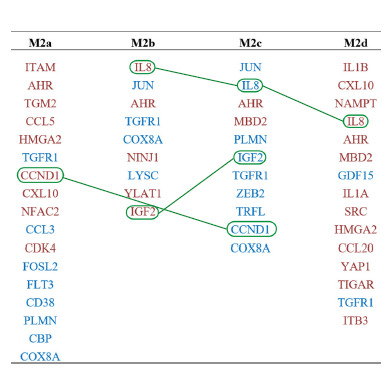
Differentially expressed proteins statistically significantly (p < 0.05) associated with the phenotype-associated genes of macrophages M2a, M2b, M2c, and M2d. Proteins, the levels of which, according to (Li et al. 2022), are elevated in a particular phenotype are highlighted in red, while those, the levels of which are decreased compared to non-activated macrophages, are highlighted in blue. Green lines connect proteins with oppositely expressed changes in macrophages of different phenotypes

Figure 4 shows the lists of differentially expressed proteins statistically significantly associated with the phenotypeassociated genes of macrophages M2a, M2b, M2c and M2d. Proteins, the levels of which are elevated in specific macrophage phenotypes according to the study (Li et al., 2022), are highlighted in red. Proteins, the levels of which are decreased compared to non-activated macrophages are highlighted in blue. Green lines connect proteins with oppositely changing expression levels in macrophages of different phenotypes.

**Fig. 5.**
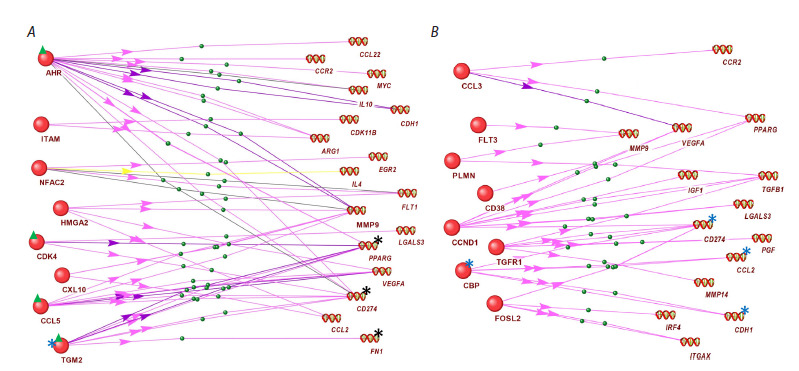
Graph of the regulation of phenotype-associated gene expression (from the MACRO_GENES database) in M2a macrophages by differentially expressed regulatory proteins from the article (Li et al., 2022), statistically significantly associated with these genes: (A) through an increase and (B) through a decrease in the level of regulatory proteins in this phenotype. The blue asterisk in (A) indicates the TGM2 protein discussed in the text, black asterisks indicate its target genes; green triangles indicate the discussed proteins AHR, CDK4, CCL2, TGM2. Blue asterisks in (B) indicate the CBP protein discussed in the text and its target genes.

Figure 5 shows examples of schemes of statistically significant regulatory interactions between differentially expressed proteins of M2a macrophages and phenotypeassociated genes reconstructed using the ANDSystem. The corresponding schemes for macrophages of the M2b, M2c, and M2d phenotypes are presented in Figures S4–S6. Figure 5 demonstrates regulatory connections using two alternative options for controlling molecular genetic processes in the same M2a macrophage phenotype: through an increase (Fig. 5A) and a decrease (Fig. 5B) in the levels of regulatory proteins. A description of the reconstructed connections obtained using frame models is given in Table 3.

**Table 3.**
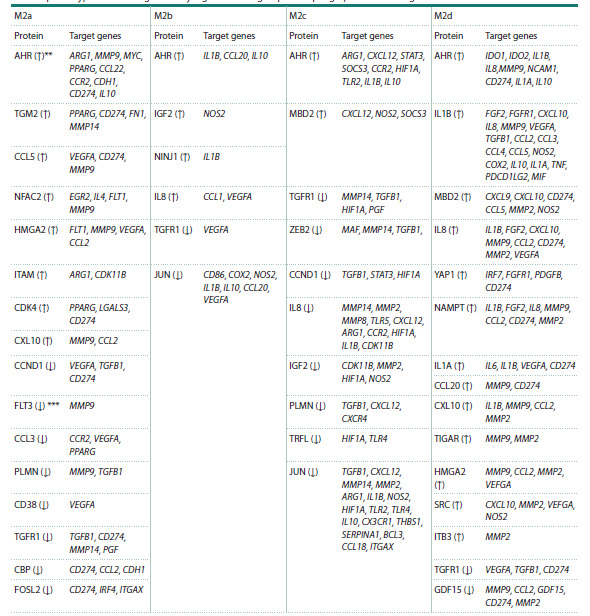
Relationships between functionally significant differentially expressed regulatory proteins* and the phenotype-associated genes they regulate in M2 group macrophages, identified using frame models * – Proteins selected based on the centrality criterion (p < 0.05); ** ↑ – proteins with increased expression levels; ***↓ – proteins with decreased expression levels.

As an example, let us consider the binding of the regulatory protein TGM2 (the lower protein in Figure 5A, marked with a blue asterisk). Proteomic data (Li et al., 2022) show that the level of this protein is elevated in the M2a phenotype compared to non-activated macrophages. According to information from the ANDSystem knowledge base obtained through its interface, in M2a macrophages, the TGM2 protein has an activating effect on the expression of the M2a phenotype-associated genes CD274 and FN1 (Liu et al., 2021; Sun et al., 2021), which is consistent with the data presented in Table 1 (the mentioned genes are marked with black asterisks in Figure 5A). TGM2 also has a suppressive effect on the PPARG gene (Maiuri et al., 2008), which is inconsistent with the data in Table 1 and indicates that the expression of this gene is also activated by some other factors, such as AHR, CDK4, CCL5, the level of which is increased in this phenotype (Fig. 5A).

Among the proteins with reduced levels (compared to non-activated macrophages) in M2a macrophages, as an example we consider the CBP protein, which regulates the phenotype- associated genes CCL2, CD274, and CDH1 (Fig. 5B, marked with asterisks). According to information from the ANDSystem knowledgebase, when CBP is suppressed in the M2a phenotype, CCL2 expression increases (Huang et al., 2021), which is consistent with the data presented in Table 1 (MACRO_GENES database). At the same time, the CBP protein positively influences the expression of the phenotypeassociated genes CD274 and CDH1 (Liu et al., 2020; Heng et al., 2021). It can be hypothesized that other regulators have a greater influence on the activity of these genes. Figure 5A shows that such regulators for the CD274 gene may include the proteins AHR, CCL5, TGM2, and CDK4, the levels of which are elevated in the M2a phenotype (Fig. 5A, double green asterisks).

All statistically significant regulatory interactions identified in M2 macrophages between differentially expressed proteins and phenotype-associated genes are presented in Table 3. For M2a macrophages, these were interactions of nine regulatory proteins with increased levels (compared to non-activated macrophages), marked with arrows (↑), and four proteins with decreased levels (↓), regulating 23 phenotype-associated genes. For M2b, these were four upregulated and two downregulated proteins regulating eight phenotype-associated genes (see also Figure S4). For M2c, two upregulated and eight downregulated proteins regulating 28 genes were identified (see also Figure S5). For M2d, 13 upregulated and two downregulated proteins regulating 29 genes were found (see also Figure S6).

Thus, based on a computer analysis of differences in the proteomes of different macrophage phenotypes, as well as the use of large volumes of information accumulated in the ANDSystem knowledge base, some regulatory proteins were identified that mediate the action of macrophage polarization inducers on phenotype-associated macrophage genes. Future research is planned using frame models containing more slots reflecting the intermediate stages of action of macrophage polarization inducers on phenotype-associated macrophage genes. This will enable the identification of more subtle features of the regulatory pathways running from macrophage polarization inducers to phenotype-associated genes through the action of intermediary proteins.

## Conclusion

A study of published data on phenotype-associated genes and proteomes of M2 macrophages, and a subsequent search for regulatory links between them using a frame-based approach implemented in the ANDSystem computer system, made it possible to identify potential regulatory proteins that mediate differences in gene expression in M2 macrophage phenotypes. The obtained results suggest that the differences between the M2a, M2b, M2c and M2d phenotypes may be associated, in particular, with the regulatory functions of the proteins JUN, IL8, NFAC2, CCND1 and YAP1, the level of which varies between phenotypes, leading to differences in the expression of phenotype-associated genes.

## Conflict of interest

The authors declare no conflict of interest.
